# EM connectomics reveals axonal target variation in a sequence-generating network

**DOI:** 10.7554/eLife.24364

**Published:** 2017-03-27

**Authors:** Jörgen Kornfeld, Sam E Benezra, Rajeevan T Narayanan, Fabian Svara, Robert Egger, Marcel Oberlaender, Winfried Denk, Michael A Long

**Affiliations:** 1 Max Planck Institute of Neurobiology, Martinsried, Germany; 2 NYU Neuroscience Institute and Department of Otolaryngology, New York University Langone Medical Center, New York, United States; 3 Center for Neural Science, New York University, New York, United States; 4 Computational Neuroanatomy Group, Max Planck Institute for Biological Cybernetics, Tübingen, Germany; 5 Bernstein Center for Computational Neuroscience, Tübingen, Germany; 6 Center of Advanced European Studies and Research, Bonn, Germany; Janelia Research Campus, Howard Hughes Medical Institute, United States

**Keywords:** connectomics, neural sequences, zebra finch, birdsong, synfire chains, Other

## Abstract

The sequential activation of neurons has been observed in various areas of the brain, but in no case is the underlying network structure well understood. Here we examined the circuit anatomy of zebra finch HVC, a cortical region that generates sequences underlying the temporal progression of the song. We combined serial block-face electron microscopy with light microscopy to determine the cell types targeted by HVC_(RA)_ neurons, which control song timing. Close to their soma, axons almost exclusively targeted inhibitory interneurons, consistent with what had been found with electrical recordings from pairs of cells. Conversely, far from the soma the targets were mostly other excitatory neurons, about half of these being other HVC_(RA)_ cells. Both observations are consistent with the notion that the neural sequences that pace the song are generated by global synaptic chains in HVC embedded within local inhibitory networks.

**DOI:** http://dx.doi.org/10.7554/eLife.24364.001

## eLife digest

For us to interact with the world around us, our brains must plan and execute our movements and behaviors. For instance, although speaking is often quite effortless, it is also remarkably complex; all of the muscles in our vocal cords have to be activated at just the right moments to create words. It remains poorly understood how exactly the brain generates such precise timing signals that enable these movements.

A specific portion of the songbird brain allows the bird to sing its song, a process that has clear parallels with human speech. Previous work had demonstrated that this region in the bird’s brain acts as a ‘clock’ for singing behavior, with individual brain cells active at just a single moment, or ‘tick’. Little consensus had been reached concerning how this might be achieved.

Kornfeld, Benezra et al. have now used new anatomical methods to better understand how the songbird clock works. A technique called 3D electron microscopy allowed the connections between the neurons in the clock brain region to be seen directly. This revealed that these neurons form direct connections with each other, which is consistent with the idea that one ‘tick’ can lead to the next and so on, like a series of falling dominoes.

Several mysteries remain to be resolved by future research. First, the connections that Kornfeld, Benezra et al. found are between cells that are quite distant from each other. This arrangement is fundamentally different from many other brain areas where neighboring cells are thought to work together. Second, although these key brain cells form appropriate connections to act as a clock, it is still not clear whether and how the network uses these connections during singing.

By resolving these mysteries, we will establish a new framework for understanding how the brain encodes learned motor gestures that may help to spur innovative new approaches for combatting motor-related deficits due to injury or disease.

**DOI:** http://dx.doi.org/10.7554/eLife.24364.002

## Introduction

**Figure 1.**
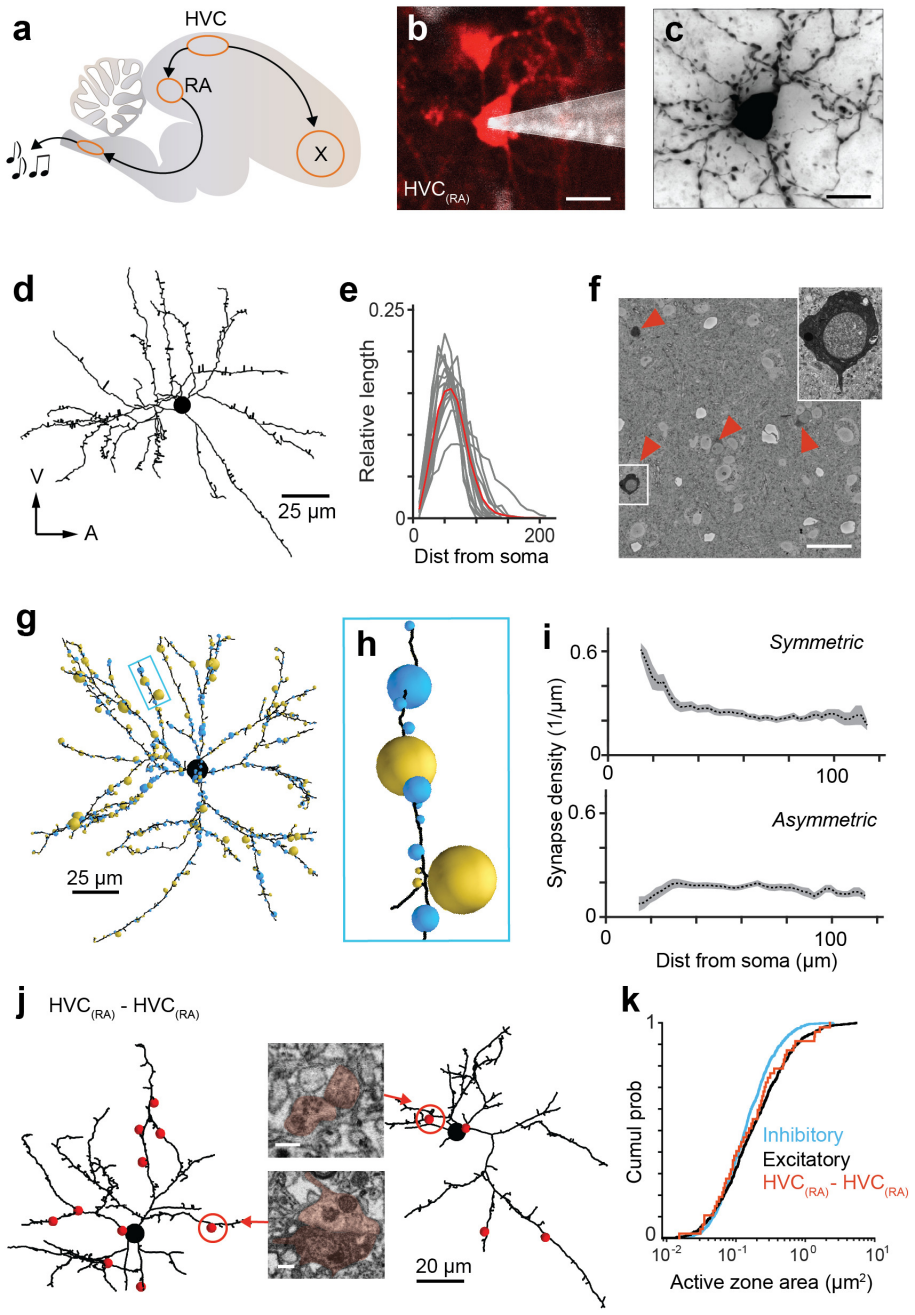
Analysis of synaptic inputs onto HVC_(RA)_ dendrites. (**a**) A schematic of the songbird brain showing HVC and its two main downstream targets, RA and Area X. (**b**) A backlabeled HVC_(RA)_ neuron (red) during juxtacellular filling (pipette shown in white) guided by 2-photon imaging of fluoro-Ruby. (**c,d**) A Neurobiotin-filled cell from (**b**) in brightfield LM after histochemical processing (**c**) and dendritic reconstruction (**d**). (**e**) Normalized count of dendritic path length vs. soma distance for 15 HVC_(RA)_ neurons; individual cells (gray) and average (red). Bin size: 10 µm. (**f**) Cross-section through a SBEM stack showing BDA-labeled HVC_(RA)_ somata. (**g**) Inhibitory (blue spheres) and excitatory (gold spheres) synapses onto an HVC_(RA)_ dendrite in the SBEM volume. Sphere cross-sectional areas are proportional to the active zone area. (**h**) Higher magnification of a dendritic branch from (**g**). (**i**) Density of asymmetric and symmetric synapses vs. the distance to the soma. (**j**) Two HVC_(RA)_ dendrites; red spheres indicate double-labeled synapses, with cross sections through two synapses (insets). Inset, cross sections through the synapses circled in red. (**k**) Active zone size distributions of inhibitory (blue), excitatory (black), and double-labeled (red) synapses. Scale bars are 10 µm in b and c, 25 µm in f, and 0.25 µm in j. **DOI:** http://dx.doi.org/10.7554/eLife.24364.003

Neural sequences are central to many models of circuit function (Diesmann et al., 1999; Jin et al., 2007; Gibb et al., 2009; Fiete et al., 2010; Mostafa and Indiveri, 2014; Cannon et al., 2015a; Rajan et al., 2016), and neurons often fire sequentially during specific behaviors (Hahnloser et al., 2002; Peters et al., 2014; Mello et al., 2015) or cognitive states (Pastalkova et al., 2008; Harvey et al., 2012), but the network properties that underlie such dynamics are poorly understood. Here we explore the synaptic connections within the zebra finch HVC, which is central to generating the neuronal activity necessary to coordinate activation of vocal muscles during the highly reproducible courtship song (Nottebohm et al., 1976; Vu et al., 1994; Aronov et al., 2008; Long and Fee, 2008). Song progression is paced by HVC_(RA)_ neurons, which project to the primary downstream target area, known as the robust nucleus of the arcopallium (RA) (Figure 1a). During the song, an HVC_(RA)_ neuron is either silent or active in the form of a burst of action potentials that occurs at a single precise and cell-specific time (Hahnloser et al., 2002; Kozhevnikov and Fee, 2007; Long et al., 2010; Vallentin and Long, 2015). At any moment, it is estimated that about 200 of these ‘pacer’ neurons are active and can drive the appropriate motor activity (Fee et al., 2004), presumably through a set of specific synaptic connections in RA (Fee et al., 2004; Markowitz et al., 2015; Lynch et al., 2016; Picardo et al., 2016).

**Figure 1—figure supplement 1.**
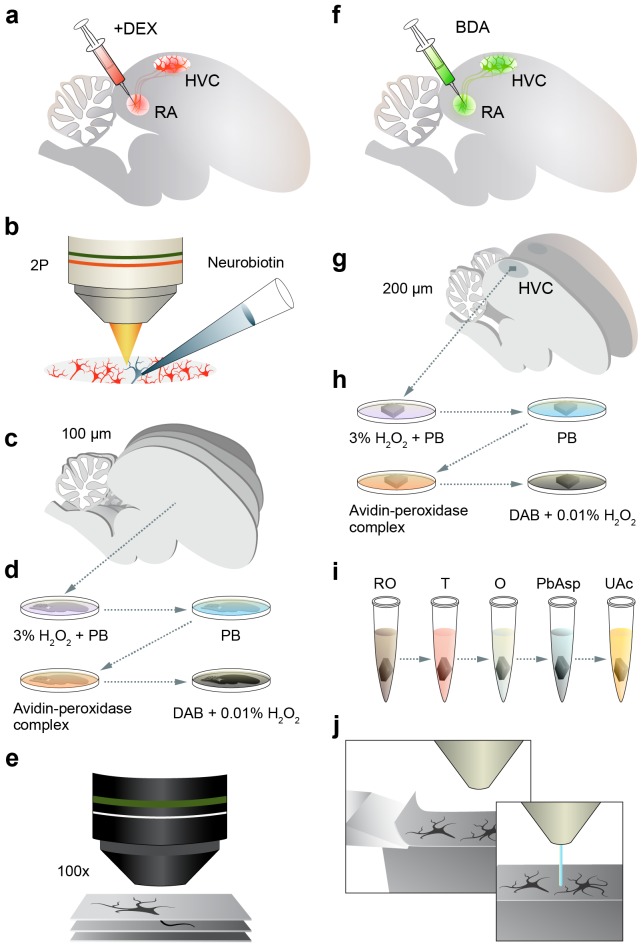
Sample preparation for LM and EM. (**a,b**) For LM, HVC_(RA)_ neurons are retrogradely labeled by injecting a fluorescent dextran (fluoro-Ruby) into RA (**a**), and a single labeled neuron is targeted and filled with Neurobiotin under the guidance of 2-photon microscopy (**b**). (**c**) The tissue is then fixed and 100 µm parasagittal sections are sliced through the entirety of HVC. (**d,e**) Sections are further processed to stain Neurobiotin-labeled neurites (**d**) and then imaged in brightfield with a 100x objective (**e**). (**f**) For EM, HVC_(RA)_ neurons are retrogradely labeled with biotinylated dextran (BDA). (**g,h**) A single 200 µm parasagittal section is taken from the center of HVC (**g**) and further processed to stain BDA-labeled neurites (**h**). (**i,j**) A cube of tissue from within the center of HVC is extracted and stained by ROTO (reduced osmium OTO) (**i**) before being imaged via SBEM (**j**). **DOI:** http://dx.doi.org/10.7554/eLife.24364.004

**Figure 1—figure supplement 2.**
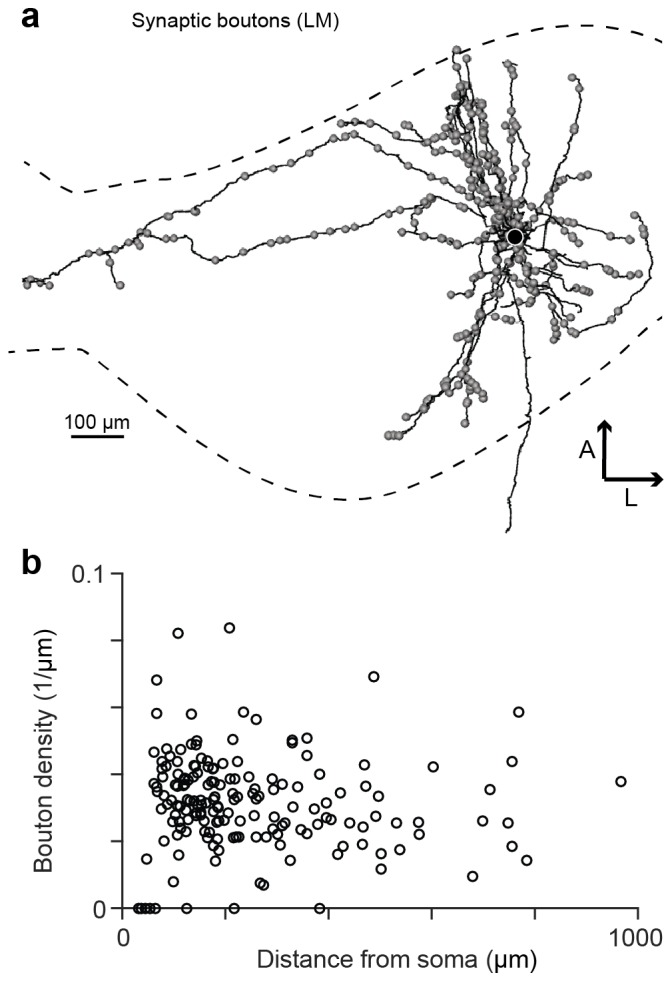
Synaptic boutons on HVC_(RA)_ axon collaterals. (**a**) An LM reconstruction of HVC_(RA)_ axon collaterals of one neuron with the HVC border indicated by dashed lines. Locations of all synaptic boutons are marked by grey spheres. (**b**) Bouton density of collateral branches as a function of their midpoint distances from the soma. **DOI:** http://dx.doi.org/10.7554/eLife.24364.005

**Figure 1—figure supplement 3.**
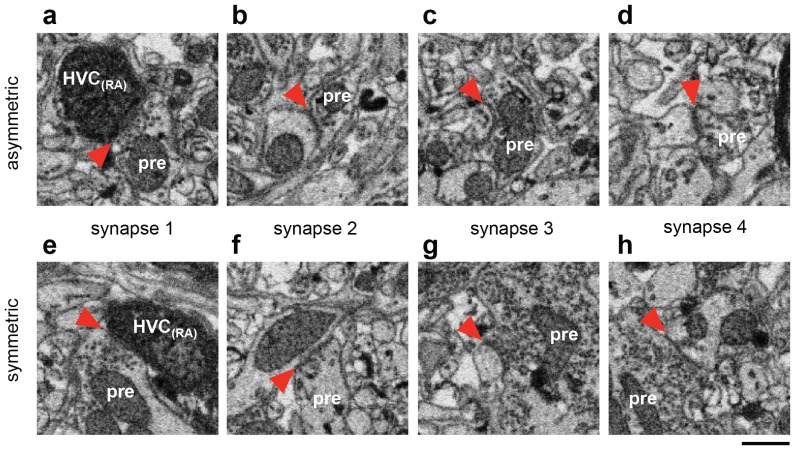
Ultrastructural classification of synapses. (**a**) An asymmetric synapse onto a BDA-labeled HVC_(RA)_ dendrite whose morphology is partially obscured by the label. Red arrows indicate the synaptic cleft. (**b** to **d**) Other synapses made by the same axon onto unlabeled dendrites. The pronounced postsynaptic density (PSD), especially for synapse 4, confirms the classification of this connection as excitatory. (**e**) A symmetric synapse onto a BDA-labeled HVC_(RA)_ dendrite. (**f** to **h**) Synapses from the same neuron onto other unlabeled dendrites display a lack of a PSD and a different appearance of synaptic vesicles compared with the asymmetric synapses. Scale bar: 0.5 µm. **DOI:** http://dx.doi.org/10.7554/eLife.24364.006

**Figure 1—figure supplement 4.**
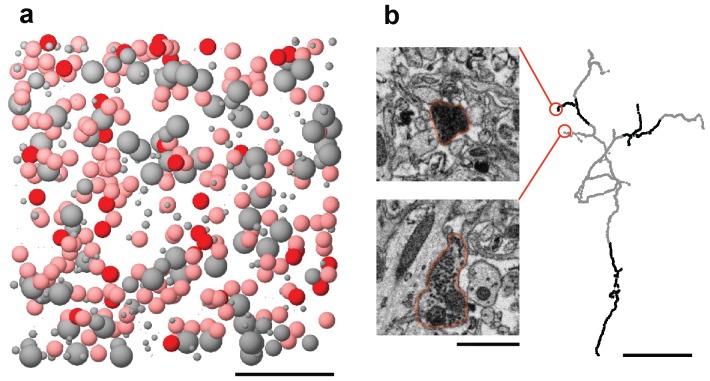
The BDA label is inefficient and incomplete. (**a**) All cells within our SBEM dataset are represented by spheres at the location of the cell body. Known HVC_(RA)_ neurons, which were labeled with BDA, were colored red. Also shown are putative HVC_(RA)_ neurons, classified by morphological features of the soma and dendrite (pink), other neuron types (large gray spheres), and glia (small gray spheres). Scale bar: 50 µm. (**b**) Incomplete labeling of axonal collaterals of HVC_(RA)_ neurons inside HVC is demonstrated with a skeleton reconstruction (labeled axon in black and unlabeled axon in gray). The inset electron micrographs correspond to the portions of the axonal field indicated. Scale bars: left: 1 µm, right: 5 µm. **DOI:** http://dx.doi.org/10.7554/eLife.24364.007

It has been difficult to discriminate between different models of sequence generation in HVC, in part because of the unknown connectivity within that nucleus. One class of models uses a synaptic (or ‘synfire’) chain architecture (Amari, 1972; Abeles, 1991; Diesmann et al., 1999), which can deliver highly reliable and precise timing but requires direct connections between the pacer neurons (Li and Greenside, 2006; Jin et al., 2007; Long et al., 2010; Cannon et al., 2015a). Such connections are, however, only rarely seen with paired intracellular recordings, which at the same time showed that HVC_(RA)_ neurons are connected with high probability (>0.50) to nearby inhibitory interneurons (Mooney and Prather, 2005; Kosche et al., 2015). This observation weakened the case for synfire chain-based sequence generation in HVC and sparked the development of alternative hypotheses that do not require direct connections between excitatory cells (Yildiz and Kiebel, 2011; Hamaguchi and Mooney, 2012; Amador et al., 2013; Goldin et al., 2013; Armstrong and Abarbanel, 2016; Hamaguchi et al., 2016; Rajan et al., 2016). There are, however, a number of reasons paired recordings may fail to correctly estimate the connection rate between excitatory cells, among them the severing of axons during slice preparation (Stepanyants et al., 2009) and an oversampling of closely spaced neurons (Jiang et al., 2015). To avoid this bias, we used a structural approach combining anatomical reconstructions of complete cells in light microscopy (LM) with high-throughput serial block-face electron microscopy (SBEM) (Denk and Horstmann, 2004; Seung, 2009).

## Results

We used both LM and EM, because anatomically, synapses can only be identified unambiguously in EM, but currently the size of the volume that can be studied by EM is limited to several hundred microns in one dimension (Helmstaedter, 2013). This size is too small to explore the full extent of HVC connectivity, given that axon collaterals of HVC neurons ramify widely throughout the nucleus (e.g. Figure 1—figure supplement 2a), which is roughly 2000 × 500 × 500 µm^3^ in size (Nixdorf-Bergweiler and Bischof, 2007). We therefore used LM to explore the mesoscale structure of the axonal morphology and EM to analyze synaptic connectivity. To identify HVC_(RA)_ cells, we injected markers into RA that are retrogradely transported, fluorescent Tetramethylrhodamine (TMR, also called fluoro-Ruby) or biotinylated dextran (BDA, Figure 1—figure supplement 1), for tissue to be observed in LM or EM, respectively.

To enable the LM-based reconstruction of the entire dendrite and of the axonal collaterals within HVC for single HVC_(RA)_ cells, we used in vivo two-photon microscopy to target (Komai et al., 2006) TMR-labeled somata for Neurobiotin labeling (Figure 1b). We eliminated all cells (29 of 44) where the labeling intensity varied between different parts of the neurite or where no descending axon could be found. The remaining 15 cells were imaged at 92 × 92 × 500 nm^3^ voxel size using a transmitted light brightfield microscope (Oberlaender et al., 2007) and reconstructed using Neuromorph (see Materials and methods) (Figure 1c,d, Figure 1—figure supplement 1a–e; Video 1). In agreement with other observations (Dutar et al., 1998; Mooney, 2000; Kosche et al., 2015), we found that HVC_(RA)_ dendrites were compact, with 95.0 ± 2.0% (SEM) of the dendritic path found within 100 µm of the soma (Figure 1e). In contrast, the axon collaterals, which were lined with synaptic boutons throughout (Figure 1—figure supplement 2b), ramified across HVC. For each cell (n = 15), the dendrite was entirely (100%) confined to HVC, while the axon (with the exception of the branch projecting to RA) was also largely restricted to the boundaries of HVC (97% on average).

**Video 1.** Video shifting through a z-stack of a sagittal section within HVC, containing a Neurobiotin-filled HVC_(RA)_ neuron stained with DAB. Number of z-sections shown is 144. Voxel size is 92 × 92 × 500 nm. **DOI:** http://dx.doi.org/10.7554/eLife.24364.008

To quantify the prevalence of different types of synaptic inputs onto the dendrite of HVC_(RA)_ cells, we next acquired a SBEM data set (166 × 166 × 77 µm^3^ overall size, comprising 15104 × 15104×2661 voxels, each 11 × 11 × 29 nm^3^ in size) from the central part of HVC (Figure 1f, Figure 1—figure supplement 1f–j, Videos 2 and 3). All raw data as well as skeletonized reconstructions are available online (Kornfeld, 2017a) (https://github.com/jmrk84/HVC_paper; with a copy archived at https://github.com/elifesciences-publications/HVC_paper). Within this volume, 34 somata were positively identified as HVC_(RA)_ neurons by the presence of a BDA-derived electron density (Figure 1f). This number is approximately 14% of the expected value of HVC_(RA)_ somata (240 ± 28, SEM), given that there are about 40,000 ± 3800 (SEM) HVC_(RA)_ cells (Wang et al., 2002) and the total HVC volume is 0.35 ± 0.024 mm^3^ (n = 14, SEM). For 12 of the 34 labeled HVC_(RA)_ neurons, we manually reconstructed (skeletonized) (Helmstaedter et al., 2011) the dendrite as far as possible. These reconstructions ranged in dendritic path length from 642 µm to 1956 µm (1290 ± 469 µm, mean ± SD) compared with complete LM-based reconstructions (1438 µm to 4819 µm, mean ± SD: 3187 ± 997 µm). Although ~70% (174 out of 248) of dendritic branches reached the boundary of the EM data set and were thus incomplete, 74 branches were completely reconstructed, including their most distal inputs (median ± SD of maximum soma distances: 90.9 ± 8.6 µm and 116.7 ± 29.4 for EM and LM, respectively). Our reconstructions therefore sample the full gamut of input types. While we do not find any variation of the input type with dendritic distance from the soma beyond a distance of 40 microns (see below), it cannot be completely ruled out that a subtle bias exists that lies below our detection threshold but might be discoverable when using larger data volumes.

**Video 2.** Video of a subregion of the acquired SBEM dataset, showing the original data resolution (lossy compression). Number of z-sections shown is 100, translating to 2.9 µm. **DOI:** http://dx.doi.org/10.7554/eLife.24364.009

**Video 3.** Video of a subregion of the acquired SBEM dataset, showing a larger field of view with a BDA-labeled HVC_(RA)_ soma (lossy compression). Number of z-sections shown is 200, translating to 5.8 µm. **DOI:** http://dx.doi.org/10.7554/eLife.24364.010

**Figure 2.**
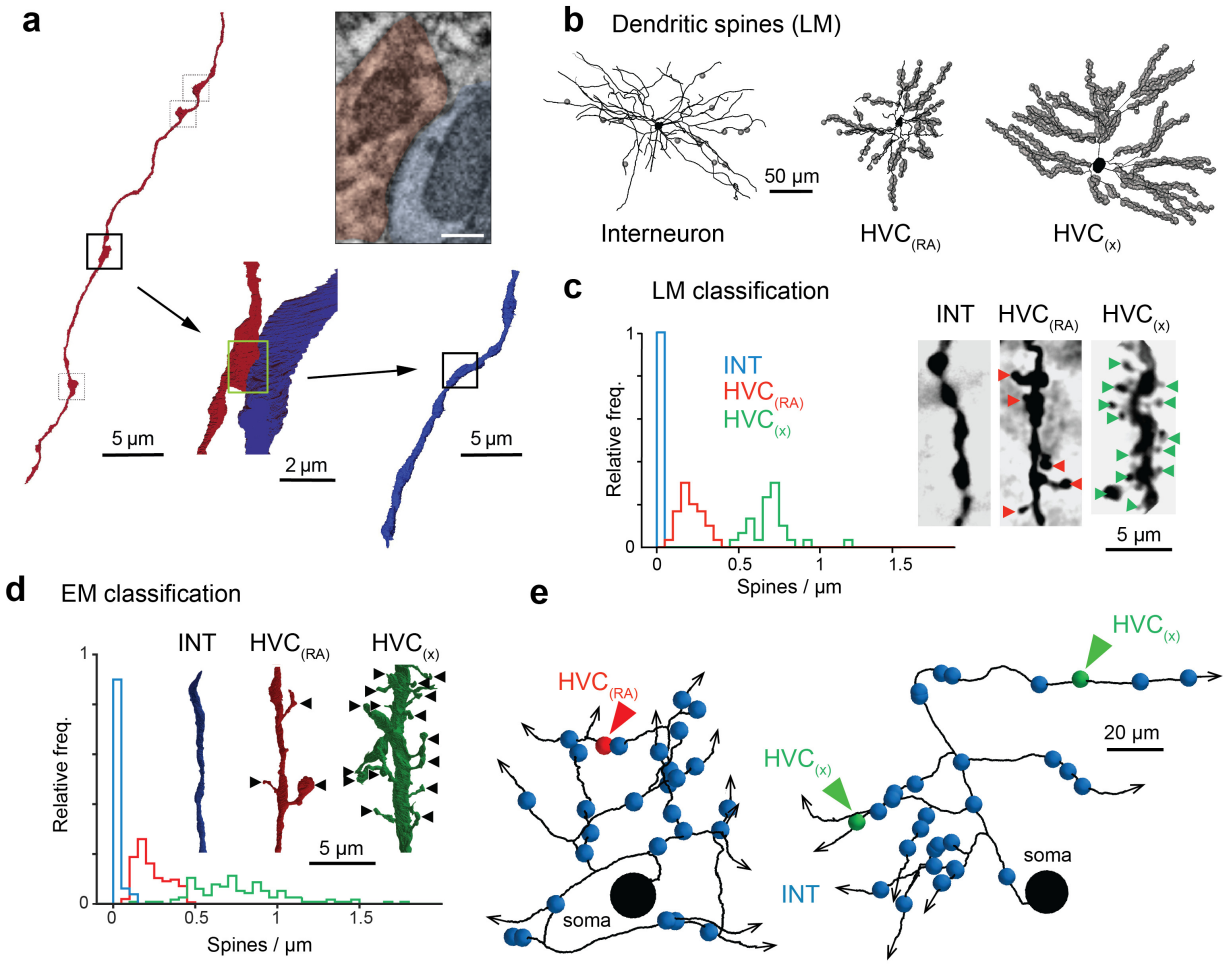
Classification of postsynaptic targets. (**a**) A BDA-labeled axon with four synaptic boutons (boxes). One bouton and its postsynaptic structure labeled in red and blue, respectively: In cross section (top right) and as a surface reconstruction (bottom center). (**b**) Dendrites from an inhibitory interneuron, an HVC_(RA)_ neuron, and an HVC_(X)_ neuron (left to right) in LM. Spine locations are indicated by grey spheres. (**c,d**) Spine densities for each of these neuron classes from LM (**c**) and EM (**d**) reconstructions. Insets show examples with spines indicated by arrowheads. (**e**) SBEM-based reconstructions of two HVC_(RA)_ somata with their proximal axons. Blue, green, and red spheres mark the location of synapses with inhibitory interneurons, HVC_(RA)_ neurons, and HVC_(X)_ neurons, respectively. Scale bar is 0.25 µm in a. **DOI:** http://dx.doi.org/10.7554/eLife.24364.011

We started by classifying for one cell all (1,003) incoming synapses (Figure 1g–h) by visually inspecting their ultrastructural details (Gray, 1959; Colonnier, 1968) (Videos 4 and 5). We found that 396 (39.5%) synapses were asymmetric and thus presumably excitatory, and 607 (60.5%) were symmetric (inhibitory) cases. If it was not possible to classify a synapse based on its inspection directly, additional synapses nearby on the same axon were analyzed, since it can be assumed that they are of the same type (Eccles, 1976) (Figure 1—figure supplement 3). Our synapse classification is reliable: in 19 out of 20 randomly selected test cases, a second expert independently came to the same conclusion and in another set of test cases (8 HVC_(RA)_, 11 HVC_(X)_, and 31 interneuron synapses), where the neuron type was known based on somatic and dendritic morphology (Figure 2d, Figure 2—figure supplement 1), all synapses were correctly classified by an expert unaware of the cell type.

**Figure 2—figure supplement 1.**
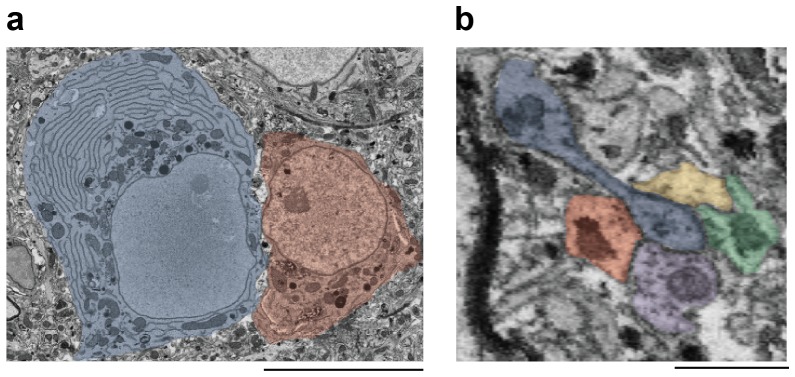
Morphological markers of interneurons. (**a**) Ultrastructural and morphological differences of the somata of an HVC interneuron (left, blue shade) and an HVC_(RA)_ neuron (right, red shade). Compared with HVC_(RA)_ neurons, interneurons had large amounts of endoplasmatic reticulum (ER), many mitochondria, and a large cell body. Scale bar: 7.5 µm. (**b**) An electron micrograph (cut plane rotated to show spine attached to dendrite) showing a polysynaptic protrusion (blue label) that receives four synapses at its tip, that was previously misclassified as a dendritic spine of an excitatory cell. The four presynaptic axons are colored in yellow, green, purple and red. Scale bar: 1 µm. **DOI:** http://dx.doi.org/10.7554/eLife.24364.012

**Video 4.** Video of a z-stack of 18 consecutive images (100 × 100 pixels) showing a symmetric synapse. Voxel dimensions: 11 × 11 × 29. **DOI:** http://dx.doi.org/10.7554/eLife.24364.013

**Video 5.** Video of a z-stack of 18 consecutive images (100 × 100 pixels) showing an asymmetric synapse. Voxel dimensions: 11 × 11 × 29. **DOI:** http://dx.doi.org/10.7554/eLife.24364.014

The dominance of inhibitory synaptic inputs was consistently observed for HVC_(RA)_ cells: when we applied our synapse classification procedure to 97 short dendritic stretches (first and third quartile of stretch length: 13.3 µm and 21.6 µm) randomly selected from eight of the other skeletonized HVC_(RA)_ cells, we found that across neurons the average ratio between excitatory and inhibitory synapses was statistically indistinguishable (p=0.36, one-way ANOVA) from that found in the completely analyzed neuron. Inhibitory synapses were significantly enriched near the soma (68 ± 4% of all synapses at most 40 µm from the soma are inhibitory compared to 57 ± 2%, for synapses beyond that distance, mean ± SEM, p<0.05, Wilcoxon rank-sum test, Figure 1i), an observation also made in cortical neurons (Anderson et al., 1994). To estimate the number of excitatory and inhibitory synapses that a single HVC_(RA)_ neuron receives on average, we first calculated dendritic synapse densities for all nine analyzed cells separately for asymmetric (0.25 ± 0.02 µm^−1^, mean ± SEM) and for symmetric synapses (0.36 ± 0.02 µm^−1^). To get expected counts per cell, we multiplied these with the full dendritic path length (on average 3.2 mm per neuron), determined from LM reconstructions. Thus, on average well above half of all synapses onto HVC_(RA)_ dendrites are symmetric (59%, 1144 ± 429, mean ± SD) and only 41% are asymmetric (786 ± 311) — a surprising dominance of inhibitory inputs that stands in stark contrast to mammalian cortical neurons (Beaulieu et al., 1992; Peters, 2002; Kasthuri et al., 2015), where the inhibitory synapses are typically found to be at most 20% of the total.

We next inspected all BDA-labeled dendrites emerging from the 12 aforementioned cells for synapses in which the presynaptic axon was labeled, and thus had to come from other HVC_(RA)_ cells (Figure 1j). We found 44 such homotypic synapses between HVC_(RA)_ cells (see Materials and methods), but they comprise only about 1% among an estimated total of 3817 ± 926 (SD) incoming excitatory synapses. Their median size (0.21 µm^2^) and size variation (first and third quartile: 0.10 µm^2^ and 0.48 µm^2^), were statistically indistinguishable from those for all asymmetric synapses (0.17 µm^2^; first and third quartile: 0.08 µm^2^ and 0.39 µm^2^, p>0.05, Wilcoxon rank-sum test, Figure 1k). One might be tempted to consider the small number of double-labeled synapses as evidence that HVC_(RA)_-HVC_(RA)_ connections are rare. However, BDA labeled only a small fraction (1/7th) of all HVC_(RA)_ cells in our data set (Figure 1—figure supplement 4a) and even for those, axonal collaterals were often incompletely filled (Figure 1—figure supplement 4b), suggesting the probability that a given stretch of HVC_(RA)_ axon is labeled could be quite small. To estimate this probability, we created a 300-member set of 1 µm^3^ cubes randomly placed throughout the SBEM volume and measured the total labeled axonal path length they contained. The value obtained (38.6 µm of labeled axon across 300 cubes) is about 13 times smaller than that expected given an estimate of the combined axonal path length (585.6 m) of all 40,000 HVC_(RA)_ cells. The axonal labeling probability of 7.6 ± 1.6% (SEM, see Materials and methods) in turn implies that the homotypic HVC_(RA)_ synapses constitute ~15 ± 4% (SEM) of all excitatory synapses onto HVC_(RA)_ neurons.

We next took a presynaptic perspective to independently estimate the extent of HVC_(RA)_-HVC_(RA)_ connectivity and used a transsynaptic tracing scheme (McGuire et al., 1991) to determine the cell-type of the targets of the outgoing synapses on BDA-labeled axon collaterals (Figure 2a). The three main cell types found in HVC (Dutar et al., 1998; Kubota and Taniguchi, 1998; Mooney, 2000) are easily distinguished in LM: Inhibitory interneurons have smooth dendrites with a nearly complete lack of spines (Mooney, 2000; Wild et al., 2005), and excitatory neurons project to either RA or to the basal ganglia (Area X), with the descending axon clearly recognizable. Even short stretches of dendrite can be reliably ascribed to one of the three types, because the spine density varies widely between but not within them (Dutar et al., 1998; Kubota and Taniguchi, 1998; Mooney, 2000) (Figure 2b,c). Dendrites were largely aspinous (0.01 ± 0.01 spines/µm, mean ± SD) for interneurons, densely covered with spines (0.70 ± 0.13 spines/µm) for HVC_(X)_ cells and less so (0.21 ± 0.07 spines/µm) for HVC_(RA)_ neurons. This spine density metric correctly classified 17 out of 18 BDA-labeled HVC_(RA)_ dendrites in EM as well as 11 inhibitory neurons that had been classified using other morphological characteristics (symmetric synapses and a large soma diameter, Figure 2—figure supplement 1a). We used this to classify the cell type of postsynaptic dendritic segments (n = 528) transsynaptically traced from nine BDA-labeled axons fully reconstructed in the EM volume. In 41 of 569 cases, the cell type could not be determined. These cases were excluded from further analysis, because the ultrastructure was obstructed by the BDA label (n = 33) or because the recovered dendritic branch was too short (n = 8), see Materials and methods, Figure 2d.

When we examined three BDA-stained axons that each emerged from labeled somata in the SBEM dataset (path lengths: 1.37, 0.88, and 0.72 mm), we found that of 121 connections, 115 terminated on dendrites of inhibitory cells but only six onto excitatory cells, four of which being other HVC_(RA)_ cells (e.g., Figure 2e). This agrees with the high connectivity found for closely spaced HVC_(RA)_-interneuron pairs by electrical recordings (Kosche et al., 2015) as well as with reports using EM connectomics for other cortical tissue (Bock et al., 2011). However, at this density, there would only be about 20 homotypic synapses per HVC_(RA)_ neuron, which is about six times smaller than our estimate derived from the BDA-labeled inputs onto HVC_(RA)_ dendrites.

**Figure 3.**
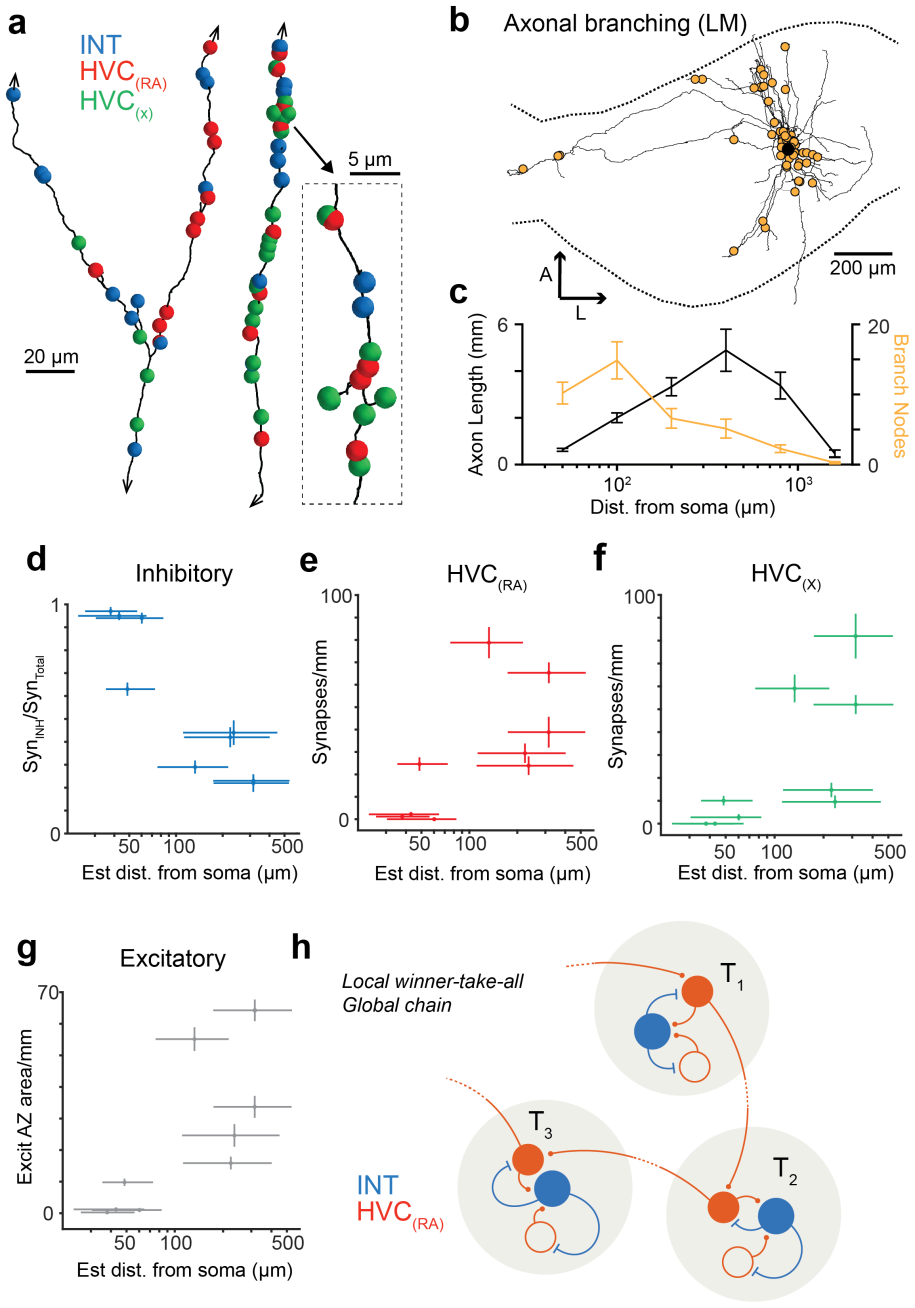
Spatial variation of postsynaptic cell type. (**a**) SBEM-based reconstructions and synaptic targets for two orphaned axon segments. Colored spheres mark the locations and types of synapses. (**b**) Axon collaterals (LM-based reconstruction) of an HVC_(RA)_ neuron with branch nodes (gold circles), the soma (black circle), and the HVC border (dashed lines). (**c**) Mean axon length (black) and branch node densities (gold) vs. soma distance (n = 15 cells). (**d**) The ratio of synapses onto inhibitory interneurons vs. estimated distance from the soma (p<0.005, Pearson's correlation). (**e,f**) The density of synapses onto HVC_(RA)_ (**e**) and HVC_(X)_ (**f**) vs. estimated distance from soma (p<0.002, Pearson's correlation, combining HVC_(RA)_ and HVC_(X)_ values). (**g**) Total synaptic size (summated active zone area, µm^2^/mm) onto excitatory neurons vs. estimated distance of the presynaptic axon from the soma (p<0.05, Pearson's correlation). Vertical error bars: SEM of the Poisson-distribution means estimated from the number of synapses on each axon segment (**e–g**) or the SEM of an assumed underlying binomial count distribution (**d**). Horizontal error bars from quantiles 0.16 to 0.84 of the distance distribution based on the nearest neighbor sampling approach (see Materials and methods). (**h**) Proposed circuit architecture. HVC_(RA)_ neurons (red) target inhibitory interneurons (blue) proximally and other HVC_(RA)_ neurons distally. **DOI:** http://dx.doi.org/10.7554/eLife.24364.015

We then examined BDA-labeled axon fragments that were 'orphaned' (n = 6, path length: 0.56 ± 0.27 mm, mean ± SD), i.e., could not be traced back to their soma and were therefore likely farther away from it. Three of the fragments were synaptically connected to one of the labeled dendrites and four were partially myelinated. We discovered that the prevalence of synapses onto excitatory neurons, and onto other HVC_(RA)_ cells in particular, was much larger for orphaned fragments than for attached axons; increases were 13-fold (HVC_(RA)_-E), from 5.0% (6 out of 121) to 64.6% (263 out of 407), and 11-fold (HVC_(RA)_-HVC_(RA)_), from 3.3% (4 out of 121) to 36.8% (150 out of 407) (Figure 3a). HVC_(RA)_ dendrites were often connected by more than one synapse to a labeled axon (17 doubles, 3 triple, and 1 quintuple among 127 analyzed pairs). The much larger (compared to the proximal outputs) fraction of excitatory target cells for the orphans implies that the prevalence of the different target types must depend on the distance from the soma. This would also be consistent with the low connection probability of 0.7% between HVC_(RA)_ cells found in electrophysiological recordings (Kosche et al., 2015), where the recorded somata are usually less than 200 µm apart (Mooney and Prather, 2005; Jiang et al., 2015), while, as our LM reconstructions show, 56 ± 14% (SD) of the axon collaterals' path lies farther than 200 µm from the soma, with some of them ramifying over the extent of HVC (e.g., Figure 1—figure supplement 2a).

**Figure 3—figure supplement 1.**
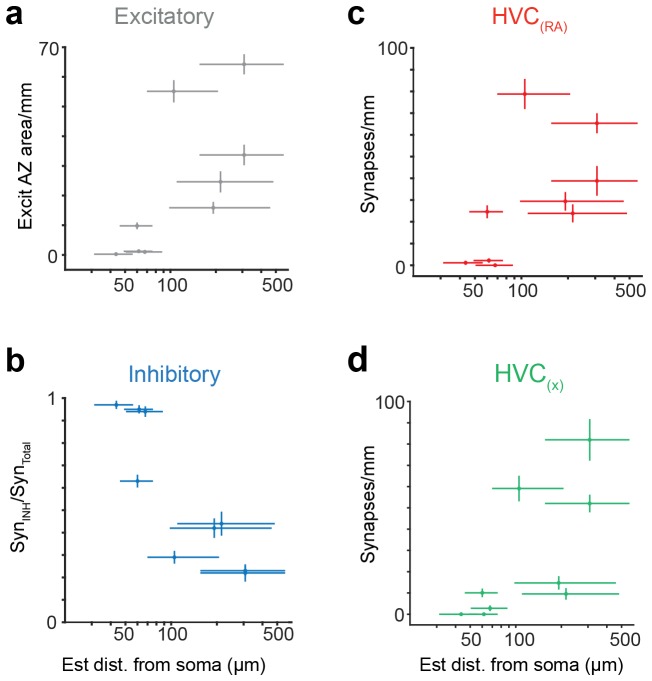
Synaptic properties of HVC_(RA)_ axons, using a Bayesian approach to estimate distance from soma. (**a**) Total synaptic strength (summated active zone area/pathlength) onto excitatory neurons vs. estimated distance of the presynaptic axon from the soma (p<0.05, Pearson's correlation). (**b**) The ratio of synapses onto inhibitory interneurons vs. estimated distance from soma (p<0.01, Pearson's correlation). (**c,d**) The density of synapses onto HVC_(RA)_ (**c**) and HVC_(X)_ (**d**) vs. estimated distance from soma (p<0.002, Pearson's correlation, combining HVC_(RA)_ and HVC_(X)_ values). Vertical bars represent the SEM of an assumed underlying Poisson distributed synapse counting process (**a,c,d**) or (**b**) the SEM of an assumed underlying Binomial count distribution. (**a–d**) Horizontal error bars correspond to a 0.16 and 0.84 quantile of the Bayesian posterior distribution, see Materials and methods. **DOI:** http://dx.doi.org/10.7554/eLife.24364.016

**Figure 3—figure supplement 2.**
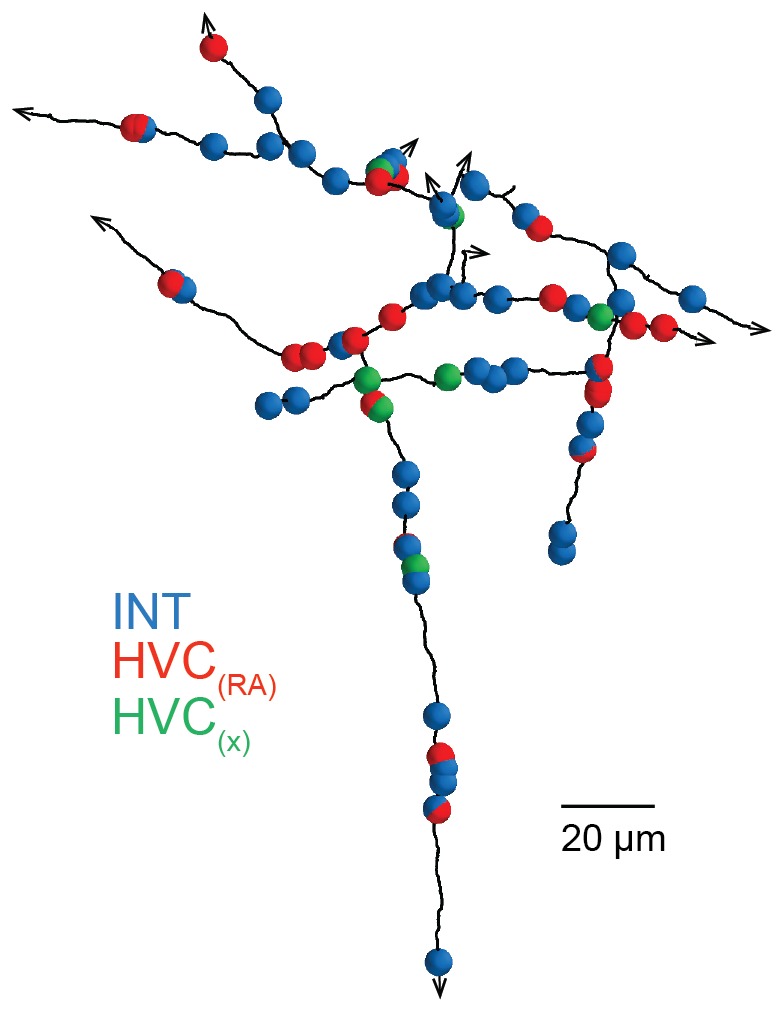
A SBEM-based reconstruction and synaptic targets for an orphaned axon with high branch density. Small spheres mark the location of synapses, with the color indicating the target type. Note the higher frequency of inhibitory targets (blue) along the length of the reconstruction compared with other orphaned axons with low branch density (see Figure 3a). **DOI:** http://dx.doi.org/10.7554/eLife.24364.017

**Figure 3—figure supplement 3.**
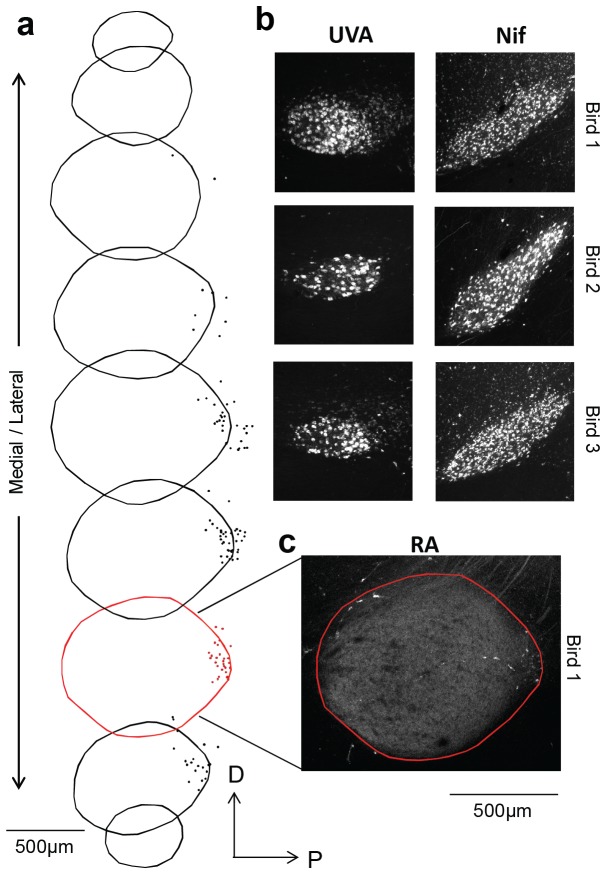
A small population of RA neurons project to HVC. (**a**) The borders of RA were traced across nine sequential 100 µm thick sagittal sections, and the location of each retrogradely labeled HVC-projecting RA neuron is marked with a dot. (**b**) DiI injection in HVC resulted in robust retrograde labeling of upstream motor nuclei Uva and NIf, while only a small percentage (<1%) of cells in RA were labeled (**a,c**). An example confocal image of RA is shown in (**c**), revealing a sparse population of retrogradely labeled neurons in the posterior region of the nucleus. **DOI:** http://dx.doi.org/10.7554/eLife.24364.018

Can we estimate the distance of an orphan segment to its soma based on local information?

It is apparent from our LM reconstructions that branching becomes less frequent as the distance from the soma increases (Figure 3b,c). Consistent with this, HVC_(RA)_ axons in the SBEM data set that were connected to a cell body were much more highly branched (12.4 ± 3.7, mean ± SD, branch points/mm, Figure 2e) than most orphaned axon fragments, with an average of only 4.0 ± 4.3 (mean ± SD) branch points/mm. To obtain a quantitative estimate of the distance to the soma and its uncertainty based on the number of branch nodes on a branch and its length we used both a nearest neighbor (Figure 3d–g) and a Bayesian (Figure 3—figure supplement 1) analysis (for details see Materials and methods). We found that a synapse was much more likely to be connected to another HVC_(RA)_ cell or to a HVC_(X)_ neuron rather than to an inhibitory neuron if the synapse was farther away from the soma (Figure 3d). The transitions between these regimes may well be gradual: One of the orphaned axons (Figure 3—figure supplement 2) showed an unusually high branch density (11.4 branch points/mm), suggesting a location close to the soma (16th to 84th percentile: 35.8 to 72.3 µm and also made the majority of its connections (52 of 83, 63%) onto interneurons, twice the fraction seen for the other orphaned axons (32 ± 10%, mean ± SD).

To rule out the possibility that our findings are due to a selection bias, we estimated the fraction of homotypic synapses for the 59 BDA-labeled axon fragments found in the 300-member set (see above), tracing each fragment from the sampling cube until we found two synapses or reached the data set boundary, and determined the postsynaptic cell types. Out of 105 synapses, 65 targeted interneurons, 22 HVC_(X)_ neurons, and 18 other HVC_(RA)_ neurons. Since there are approximately 1111 ± 513 (SD) outgoing synapses inside HVC for each HVC_(RA)_ neuron (given an axon path length of 14.7 mm and a total synapse density of 75.4 synapses/mm), we expect about ~191 ± 88 (SD) homotypic synapses per cell (on average, incoming and outgoing homotypic synapse have to be equal in number), comprising about a quarter (24 ± 4%, SEM) of all incoming excitatory synapses, and nearly half of all outgoing excitatory contacts. The discrepancy between the estimates of the homotypic fraction of incoming excitatory synapses from the dendritic (~15%) and axonal perspective (~24%) might be due to the fact that when counting the number of double labeled synapses, we accepted only those where the labeling of the presynaptic terminal was unambiguous.

How can we be sure that all or at least most of the orphaned fragments belong to HVC_(RA)_ neurons? Since BDA (which is transported in the retrograde direction much more efficiently than anterogradely in all tissues tested, including the zebra finch brain (Reiner et al., 2000) was only injected into RA, any labeled axon has to belong to a cell with an axon that connects HVC and RA, as HVC_(RA)_ axons do. If there is indeed a substantial number of cells in RA that project to HVC(Roberts et al., 2008), then it is possible that a substantial fraction of the orphaned axons could originate from those cells. To independently confirm the number of RA_(HVC)_ cells, we injected the fluorescent tracer DiI (Invitrogen, Carlsbad, CA) into HVC, which heavily labeled the upstream nuclei NIf and Uva (Figure 3—figure supplement 3) but yielded only a small number of labeled somata in RA (125, 163, and 171, respectively, in three birds), approximately one for every 200 HVC_(RA)_ neurons on average. To account for the density of labeled axon in our EM volume, each those cells would need a total axon path of ~4 m in HVC, which appears unlikely given that the extensively ramifying HVC_(RA)_ axons have a length of only ~0.015 m.

## Discussion

We have shown that the synaptic architecture in HVC contains a density of connections between HVC_(RA)_ neurons that might be sufficient to support a synaptic-chain model, whereby precisely timed sequences of action potential bursts in HVC_(RA)_ neurons are generated by a wave of activity propagating via synaptic connections among these neurons without the need for inhibition-mediated propagation of activity (Yildiz and Kiebel, 2011) or to involve structures outside HVC (Hamaguchi and Mooney, 2012; Goldin et al., 2013; Hamaguchi et al., 2016).

While we estimate that 25% of excitatory inputs to HVC_(RA)_ neurons are homotypic, the sources of the remaining synapses are unknown. It should be a central goal of future efforts to quantify the relative number of connections from these regions (e.g., Uva, NIf, etc.) at the level of single HVC_(RA)_ neurons. That said, many of these connections, such as auditory afferents (Vallentin and Long, 2015), collaterals from HVC_(X)_ neurons (Scharff et al., 2000), and descending fibers from NIf are unlikely to play a role in motor patterning, since removal of NIf does not disrupt the song (Cardin et al., 2005). The precise role of Uva, a thalamic region also directly projecting to HVC (Nottebohm et al., 1982), remains to be determined (Coleman and Vu, 2005; Hamaguchi et al., 2016).

Somewhat surprisingly, the low rates of pairwise connectivity seen in electrophysiological recordings (Mooney and Prather, 2005; Kosche et al., 2015), which previously had been interpreted as evidence against a direct synaptic chain (Armstrong and Abarbanel, 2016), are not inconsistent with our estimate that each HVC_(RA)_ neuron receives a significant amount of its excitatory input from other HVC_(RA)_ neurons. The reason is that with the around 200 homotypic inputs per cell, the probability to be connected to any one of around 40,000 HVC_(RA)_ neurons can be at most 0.5%. The question remains what fraction of those inputs are true ‘chain’ synapses in that the presynaptic cell’s activity immediately precedes that of the postsynaptic cell, but our study demonstrates that the anatomical substrate for the chain model exists.

An important next step will be to combine functional imaging with volume EM to directly test whether an HVC_(RA)_ cell receives more numerous or stronger direct homotypic inputs from cells that fire immediately prior to its own activity. In fact, a recent study describes how calcium activity can be imaged in the singing bird (Picardo et al., 2016), a crucial step in that direction. One potential difficulty stems from our finding that HVC_(RA)_ neurons preferentially form distal connections, indicating that the timing circuitry in HVC is distributed and therefore requires a large EM volume (as much as 500 million µm^3^, compared to 2 million µm^3^ in our volume) for its complete reconstruction. It might take the better part of a year merely to acquire the raw data (Schalek et al., 2016). While even a few years ago it seemed impossible to analyze such an amount of data within a reasonable time frame, recent progress in the automation of segmentation are encouraging (Berning et al., 2015; Januszewski et al., 2016; Beier et al., 2017; Dorkenwald et al., 2017).

Our finding that connections near the soma are often onto inhibitory neurons suggests that inhibition plays an important role in sequence generation, which is further supported by the large overall fraction of inhibitory inputs. One function of those inhibitory connections could be to decorrelate excitatory activity in space and time: Not only are nearby HVC_(RA)_ neurons rarely connected and thus unable to drive each other, but even when driven by a common input, only the cell(s) with the strongest input(s) will continue to fire in the face of the winner-take-all effect due to the strong reciprocal inhibition (Figure 3h). Winner-take-all behavior is normally associated with certain cognitive tasks (Hopfield and Tank, 1985; Lundqvist et al., 2016), such as decision making (Usher and McClelland, 2001). In HVC, it may help to prevent local clusters of activity, which could lead to leakage across different chains passing through adjacent excitatory neurons. An altogether different role for local inhibition may be the improvement of temporal precision by sharpening burst timing through recurrent inhibition (Hahnloser et al., 2002; Long et al., 2010; Cannon et al., 2015a).

Inhibition may, furthermore, have a central role in shaping the distance dependency of postsynaptic targets during circuit development without the need for molecular cues (de Wit and Ghosh, 2016). Instead, the architecture we observed may arise naturally from a pattern that initially follows Peters' rule (Braitenberg and Schüz, 1998), which predicts synaptic connections between cell types with intermingled axonal and dendritic arbors (Rees et al., 2017), but is then refined as the interneurons increasingly prevent the co-activation of nearby excitatory cells, thereby destabilizing connections between them while leaving more distant connections intact. Such a preferentially distal connectivity would also favor more widely distributed synaptic chains, which could have the added benefit of relying more on axonal propagation delays for sequence timing (Budd et al., 2010). Overall, the observed synaptic architecture shows some resemblance with local inhibitory/excitatory networks linked by long-range excitatory/excitatory connections (coupled winner-take-all modules) that have been shown to make computational models of cortical sequence generation more robust (Binas et al., 2014; Mostafa and Indiveri, 2014).

## Materials and methods

### Animals

We used adult (>90 days post hatch) male zebra finches that were obtained from an outside breeder and maintained in a temperature- and humidity-controlled environment with a 12/12 hr light/dark schedule. All animal maintenance and experimental procedures were performed according to the guidelines established by the Institutional Animal Care and Use Committee at the New York University Langone Medical Center.

### Surgery

To label only neurons that projected from HVC to the robust nucleus of the arcopallium (RA), we injected lysine-fixable retrograde dextran tracers (Invitrogen) conjugated to either Tetramethylrhodamine (fluoro-Ruby, mol. Weight: 10,000) or biotin (BDA, mol. weight: 3000) for preparations to be inspected with light microscopy (LM) or electron microscopy (EM), respectively. We injected 200 nL of either Fluoro-Ruby (50 mg/mL) or BDA (100 mg/mL) into RA of anesthetized (1–3% isoflurane in oxygen) zebra finches using an injection system (Nanoject, Drummond Scientific, Broomall, PA) outfitted with a glass injection pipette (tip diameter: 30–40 µm). RA was targeted using stereotaxic coordinates (2.30 mm lateral and 1.85 mm posterior from the midsagittal sinus) and success in finding the RA region was confirmed by observing characteristic spontaneous activity (Long and Fee, 2008) using a carbon-fiber electrode (Carbostar-1, Kation Scientific, Minneapolis, MN) and an extracellular amplifier (NPI Electronic Instruments, Germany).

For in vivo imaging and dye loading, we first had to enable optical access to HVC. To accomplish this, a craniotomy (1 mm x 1 mm) was prepared over HVC. The underlying dura was then carefully removed with a flame sharpened tungsten wire (starting diameter: 0.5 mm). A small drop of saline buffer was applied to the exposed brain, followed by a 3 mm-diameter round cover glass (#0 thickness, Warner Instruments, Hamden, CT) as an optical window, which was first secured to the surrounding skull by applying light-curable acrylic (Flow-IT ALC; Pentron Clinical Technologies) around the edges of the glass. Dental acrylic (Cooralite Dental MFG, Diamond Springs, CA) and cyanoacrylate were then added to permanently and stably attach the cover glass to the skull. A small metal head plate with two tapped holes was then implanted at the anterior part of the skull using dental acrylic for head fixation.

### 2-Photon guided cell labeling

Juxtacellular labeling (Pinault, 1996; Narayanan et al., 2015) with Neurobiotin (Vector Labs, Burlingame, CA) was used to fill individual RA-projecting HVC (HVC_(RA)_) neurons out of a population that had been retrogradely labeled from RA with fluoro-Ruby in vivo. After waiting at least 48 hr following the injection of the retrograde tracer into RA, two-photon imaging (Denk and Webb, 1990) was used to identify the target cell and guide the pipette. On the day of single-cell labeling, a small pipette access hole (~400–500 µm) was drilled in the glass coverslip immediately lateral to the target recording region using a carbide bur drill bit (1/4 FG-100; Johnson-Promident). Glass pipettes were fabricated using a horizontal puller (P97, Sutter Instrument Company, Novato, CA) and had a final resistance of 4–5 MΩ when loaded with internal solution that consisted of 150 mM K-Gluconate (Sigma-Aldrich, St. Louis, MO) and 3% Neurobiotin. The microscope (MOM, Sutter Instrument Company) was of the moveable objective design (Euler et al., 2009) and was controlled using ScanImage (Pologruto et al., 2003) 3.8 with a 16x/0.8 NA water immersion objective (Nikon, Japan). Pipettes were made fluorescent either by adding 40 µM of Alexa 488 (Invitrogen) to the internal solution or by coating the pipette with green fluorescent quantum dots (Andrásfalvy et al., 2014). The activity of HVC_(RA)_ neurons was recorded (IR-183, Cygnus Technology Inc, Delaware Water Gap, PA), and cells were filled with Neurobiotin by applying 1000–1500 positive current pulses with an amplitude between 3 and 15 nA and a duration of 200 ms delivered at a frequency of 2.5 Hz.

### Histological procedures (LM)

Birds were anesthetized with pentobarbital sodium and perfused transcardially with 4% w/v paraformaldehyde (EMS) at least one hour after dye loading to permit adequate Neurobiotin diffusion. Brains were removed from the skull using a surgical scoop, immersed in 4% paraformaldehyde for 3–5 days to achieve thorough fixation, and incubated in phosphate buffer for an additional 1–3 days to decrease endogenous peroxidase activity. To prepare sections, the brain was cut across the midline, mounted on the sagittal surface with cyanoacrylate, and stabilized with 3% agarose. Parasagittal sections (100 µm thickness) of HVC were cut using a vibratome (Leica VT1000S). Slices were washed five times with phosphate buffer and treated with 3% H_2_O_2_ to further reduce endogenous peroxidase activity. Slices were then immersed overnight at 4°C in a solution containing avidin/biotin complexes and 0.5% Triton X-100 in phosphate buffer (Vector Labs and Sigma-Aldrich, respectively) to tag the Neurobiotin with peroxidase complex. On the following day, slices were washed five times with phosphate buffer and then immersed in a solution containing 2.3 mM diaminobenzidine (DAB, Sigma-Aldrich) and 0.01% H_2_O_2_ in phosphate buffer to label processes containing Neurobiotin. Slices were then washed and mounted on slides with Vectashield (Vector Labs) or Mowiol (Sigma-Aldrich) mounting medium.

To quantify the number of HVC-projecting RA neurons, we injected a retrograde tracer into HVC (DiI, Invitrogen D3911; 46 nL total injection volume) that labels neurons with high efficiency in zebra finches (Scott et al., 2012). Following a two-day incubation period, animals were perfused with 4% paraformaldehyde, and 100 µm sagittal sections were cut across the entirety of RA, Nucleus Interfacialis (NIf), and nucleus Uvaeformis (Uva). Sections were mounted on slides using Vectashield (Vector Labs) and imaged with a confocal microscope (LSM 800, Zeiss, Germany; excitation / emission: 551/569 nm) using a 20x objective (0.8 NA). The z-stacks of retrogradely labeled RA_(HVC)_ neurons were captured across the extent of RA, and the position of each cell was manually marked using the landmark function in Amira.

### LM imaging

Only well-filled HVC_(RA)_ neurons were selected for reconstruction, specifically those in which the soma, dendrite, and axon were all labeled (even if faintly) without interruptions and with clearly labeled dendritic spines and presynaptic boutons were selected for high resolution LM imaging with a custom-designed high-resolution mosaic/optical-sectioning brightfield microscope system (Oberlaender et al., 2007). In brief, a transmitted light brightfield microscope (Olympus BX51, Olympus, Japan), equipped with a motorized x-y-z stage (Maerzhaeuser, Germany), a narrow bandpass (546 ± 5 nm) illumination filter and a 100x magnification oil-immersion objective (numerical aperture 1.4) was used to acquire image stacks from consecutive 100 µm thick brain sections. For each section, a 3D mosaic of images (e.g., 10 × 15 fields of view) covering the entire HVC was acquired at 92 × 92 nm pixel size and in steps of 500 nm mechanical defocus. Next we applied a linear image restoration algorithm (Tikhonow-Miller) using the Huygens software package (Scientific Volume Imaging, Netherlands). By inverting the gray values of the brightfield image stacks they could be treated as fluorescent data with an emission wavelength of 546 nm. The deconvolution used a point-spread-function that takes the optical properties of biocytin-labeled brain tissue into account (Oberlaender et al., 2009). Deconvolved image stacks were then downsampled by a factor of two in x/y, yielding a final voxel size of 184 × 184 × 500 nm before axonal reconstruction. To quantify the bouton density, subvolumes that contained primarily horizontal (i.e. within the image plane) axonal branches were acquired at 200 nm focus increments and used without deconvolution.

### Neuron reconstructions (LM)

Neuronal branches (dendrites and axons) were reconstructed in 3D using NeuroMorph (Oberlaender et al., 2007). Automated tracing results from each histological section were manually proof-edited using FilamentEditor (Dercksen et al., 2014), custom-designed based on Amira visualization software (FEI-VisualizationSciencesGroup). In brief, maximum-intensity z-projections of the original image stacks were superimposed onto automatically generated 3D skeleton tracings of all putative neuronal branches contained within the imaged volume and segmented objects that had no correspondence in the projection image were manually deleted (Dercksen et al., 2014). Fragmented segments were spliced, and axonal branches were classified as ‘dendrite’ or ‘axon’ based on whether, respectively, spines or boutons were visible in the projection images. Whenever a neuronal branch reached one of the borders of the imaged volume, additional image stack regions were acquired that allowed us to follow the branch further. To account for shrinkage during histological processing, the reconstruction was scaled to match the thickness of 100 µm, as defined by the vibratome. The scaled 3D tracings from all consecutive sections were then combined and manually aligned using the FilamentEditor. The z-coordinate of each point was then replaced by the average of nine points (the point itself and the four adjacent points in each direction) and resampled to a point spacing of about 1 µm. Smoothing in z and downsampling make path length measurements comparable to manual tracing results using Neurolucida Software (Microbrightfield, Williston, VT). The NeuroMorph and FilamentEditor tools enable tracings that are independent of the experience of the human operator, with an interuser-variability of approximately 20 µm per 1 mm axonal length(Dercksen et al., 2014). The borders of HVC were manually traced in each 100 µm tissue section using Neurolucida.

### Analysis of LM reconstructions

The fraction of dendritic length contained within a certain distance of the soma was determined by conducting a spherical Sholl analysis (Sholl, 1953) in Neurolucida (Microbrightfield). The proportion of axonal pathlength both within HVC and within a 200 µm radius from the soma was computed in Amira for each neuron using the ZIB extension package (Egger et al., 2014). Axonal boutons and dendritic spines were annotated manually in Amira using high-resolution LM stacks. The location of each bouton or spine was marked in 3D and aligned in Amira to the corresponding branch reconstruction. Spine-densities were calculated for each branch by dividing its total spine count by its path length. Branch nodes (points where the axon bifurcates) were manually located in the reconstructions using Amira. Branch nodes for which one of the daughter branches was <15 µm in length were not included in this analysis.

### Histological procedures (EM)

The bird used for the EM experiments was transcardially perfused in a way that preserves the extracellular space and leads to minimal shrinkage (JK, unpublished observations), by using high pressure and the following fixative solution: 0.07 M sodium cacodylate (Serva, Germany), 140 M sucrose (Sigma-Aldrich), 2 mM CaCl_2_ (Sigma-Aldrich) with 2% paraformaldehyde and 2% glutaraldehyde (Serva) added (Cragg, 1980). The brain was removed and, using a vibratome (Leica VT1000S), cut into slices each about 200 µm thick. One of the slabs that centrally intersected HVC was selected and post-fixed in the same solution overnight, rinsed several times with cacodylate buffer and permeabilized in a 30% sucrose solution by exposing it to one freeze-thaw cycle in liquid nitrogen. Residual peroxidase activity was suppressed by soaking the sample in 3% H_2_O_2_ for 30 min before labeling the sample with an avidin-peroxidase complex and DAB, as described in a previous section. The sample was then rinsed several times in cacodylate buffer. Heavy metal staining was added through a conventional ROTO protocol using the following steps interspersed with rinses in cacodylate buffer (after first Osmium step) or H_2_O (all others): 2% OsO_4_ (Serva), reduced with 2.5% potassium hexacyanoferrate(II) (Sigma-Aldrich) 2 hr, room temperature; 1% thiocarbohydrazide in H_2_O, 1 hr, 58°C (Sigma-Aldrich); 2% OsO_4_, 2 hr; 1.5% uranyl-acetate in H_2_O, 53°C (Serva); 20 mM lead-aspartate, 2 hr, 53°C (Sigma-Aldrich) (Seligman et al., 1966; Karnovsky, 1971; Walton, 1979). Dehydration was performed using an ethanol series with 10, 15, 10, 10 min at 70%, 100%, 100%, and 100% ethanol (Electron Microscopy Sciences). The sample was infiltrated with epoxy monomer (epon hard, Serva) (Glauert and Lewis, 2014) dissolved in propylene oxide (Sigma-Aldrich) for 3 hr and for 3 hr with pure monomer before final embedding and curing (48 hr at 60°C). The sample was then trimmed and glued with epoxy to a custom-made aluminum holder and trimmed into a pyramidal-shape before gold coating for better conductivity.

### SBEM imaging and data preprocessing

We performed serial block-face electron microscopy (Denk and Horstmann, 2004) at 11 × 11 × 29 nm voxel size using a scanning electron microscope with a field-emission cathode (UltraPlus, Zeiss, Germany) equipped with a custom-built in-chamber microtome in high-vacuum (raw and effective voxel rates were 5 and 2.1 MHz respectively) at a dose of 10.3 electrons/nm^2^, 2 kV landing energy with a custom back-scatter electron detector and amplification system optimized for fast acquisition speeds. Before each cut, a subregion of the block face was imaged using four overlapping micrographs resulting in an image stack. Images were registered by affine transformations (https://github.com/billkarsh/Alignment_Projects) (Scheffer et al., 2013; Karsh, 2016) and converted to a KNOSSOS (www.knossostool.org) data set for reconstruction and browsing with custom Python code (https://github.com/knossos-project/knossos_python_tools/tree/master/knossos_cuber) (Kornfeld, 2017b). Copies of the software are archived at https://github.com/elifesciences-publications/Alignment_Projects and https://github.com/elifesciences-publications/knossos_utils).

### Neuron reconstructions (EM)

Each annotator received at least 10 hr of training and was considered an expert after one year of annotation experience. BDA-labeled neurons, using the soma as a starting place, were skeletonized within the EM stack in KNOSSOS by an expert annotator, and errors were corrected by the same individual in a second pass, which was also used for synapse annotation. All BDA-labeled axons, including orphaned axons, were traced by at least two independent annotators and discrepancies were resolved by an expert that had not participated in the initial annotation. Synapses on each axon were then labeled (see synapse identification) and proofread by an expert annotator who excluded cases where the BDA-label obscured the ultrastructure. The remaining synapses were used to seed the tracing of the postsynaptic dendrite segment. Annotators were instructed to reconstruct the postsynaptic dendrite to the end of the branch in one direction and to the next main branch point in the other direction. All dendritic-branch tracings were proofread by an expert and only included if at least a minimum path length of 10 µm could be reconstructed. All EM reconstructions were analyzed and visualized with custom Python code using the Mayavi2 (Enthought) library (Kornfeld, 2017a, Kornfeld, 2017b).

### EM synapse annotation

Synapses were labeled by an expert annotator and classified as symmetric or asymmetric (Videos 4 and 5, Figure 1—figure supplement 3). Active-zone’ diameters ‘were quantified by measuring - with KNOSSOS - the cross-sectional length of the synaptic thickening in that plane and principal viewing orientation (x, y, or z) in which the contact cross section appeared largest. Diameters were then converted to areas by assuming a circular synaptic contact.

### Classification of postsynaptic cell type

To estimate dendritic spine density, a stretch of the postsynaptic dendrite (>10 µm) was selected that often included the place where the axon was in contact with the dendrite. We counted as a spine every skeleton branch with a length greater than 1 µm that emerged from the dendritic shaft. Some postsynaptic protrusions found on interneurons contained multiple synapses (e.g., Figure 2—figure supplement 1b). Therefore, spines were defined as receiving no more than one synapse at their ends by three independent annotators. The resulting spine density D_spine_ (in µm^−1^) was used to classify the dendritic stretch as belonging to an interneuron (D_spine_ < 0.11), HVC_(RA)_ (0.11 < D_spine_ < 0.46), or HVC_(X)_ neuron (0.46 < D_spine_). To detect dendritic reconstructions that were traced from separate synapses but belonged to the same dendrite, we detected overlap between skeletons using the following criterion: a node was considered to overlap another skeleton if it was less the 400 nm from any edge of all other skeletons. Dendritic reconstructions were defined as belonging to the same neuron when at least 25% of their nodes overlapped. Since the postsynaptic dendritic reconstructions were never complete (i.e. only parts of the entire neuron could be reconstructed), our analysis could only positively identify reconstructions as belonging to the same cell. For dendrites that were found to belong to the same cell (grouped together after being traced from different synapses), spine density was averaged before classification.

### Estimating the axon-to-soma distance

We used two different ways to estimate the distance between an orphaned branch and its soma from its number of branch nodes inside the EM volume, both based on the LM observation that the density of branch nodes, D_b_, varies with soma distance (r) (Figure 3c). The first way used a Bayesian approach to calculate the probability distribution over r, given a branch of length *l* and a branch-node count of *N* (Figure 3c), which can be used to estimate, as needed, mean, median, variance or any quantile for r:

$$P\left(r\text{|}N,l\right)\propto P\left(N,l\text{|}r\right){*P}_{a}\left(r\right),$$

whereby

$$P\left(N,l\text{|}r\right)=\frac{{\left({\text{D}}_{b}\left(r\right)\text{*}l\right)}^{N}\text{*}e^{-D_{b}\left(r\right)\text{*}l}}{N!},$$

which assumes that the branch nodes are placed independently from each other and are, therefore, Poisson distributed with a node-count expectation value of $\lambda =D_{b}\left(r\right)\text{*}l$. Fitting the LM measurements to an exponential gave $D_{b}\left(r\right)=\left(35.448\text{*}e^{-\frac{r}{43.5mm}}+0.613\right)\text{mm}$. The Bayesian prior, $P_{a}(r)$, i.e. the probability that an axon segment is found at a distance between r and r±� from its soma, was estimated by applying Gaussian kernel density estimation (Python scipy.stats.gaussian_kde, scott bandwidth selector) to the LM based axon distribution measurements.

The other way to relate r to N and l is to sample the LM data directly: We divided each of the 15 LM stacks into volumes shaped identically to the EM volume and recorded for each volume and for all contained orphaned branches their lengths, distances from the soma, and branch-node counts. Only branches that both entered and left the sampled subvolume were considered (about 95% of the total) because all of the reconstructed orphaned branches in the EM volume also had that property. This was repeated with the origin of the division grid shifted in 10 µm increments along all three axes resulting in 17 × 17 × 8 different divisions for each LM stack. For a given orphaned branch in the EM volume, we selected all those sample branches that had the same node count and a length within ± 10%. The distribution of their soma distances was then used in the same way as the probability distribution coming from the Bayesian approach.

### Estimating the fraction of homotypic HVC_(RA)_ synapses

In order to estimate the homotypic fraction of all excitatory synapses onto HVC_(RA)_ cells, we determined the density of homotypic synapses by counting the number of double labeled synapses and correcting it for the axonal labeling efficiency. Labeling efficiency was estimated by comparing the volume density of labeled axon length by inspecting 300 randomly placed 1 µm^3^ cubes with the density expected for HVC_(RA)_ neurons using published estimates for their total number (Wang et al., 2002) and the average axonal path length from LM reconstructions. To count the number of double labeled synapses, BDA-labeled dendrites were searched by an expert annotator for synapses with labeled axons by following them in KNOSSOS at the full voxel resolution, instructed to annotate also synapses with weak labeling. The found synapses were then scrutinized by JK and the result was confirmed by ML and SB.

All error estimates were calculated assuming independence of the errors using the variance formula for error propagation.

## References

[bib1] Abeles M (1991). Corticonics: Neural Circuts of the Cerebral Cortex.

[bib2] Amador A, Perl YS, Mindlin GB, Margoliash D (2013). Elemental gesture dynamics are encoded by song premotor cortical neurons. Nature.

[bib3] Amari S-I (1972). Learning patterns and pattern sequences by self-organizing nets of threshold elements. IEEE Transactions on Computers.

[bib4] Anderson JC, Douglas RJ, Martin KA, Nelson JC (1994). Map of the synapses formed with the dendrites of spiny stellate neurons of cat visual cortex. The Journal of Comparative Neurology.

[bib5] Andrásfalvy BK, Galiñanes GL, Huber D, Barbic M, Macklin JJ, Susumu K, Delehanty JB, Huston AL, Makara JK, Medintz IL (2014). Quantum dot-based multiphoton fluorescent pipettes for targeted neuronal electrophysiology. Nature Methods.

[bib6] Armstrong E, Abarbanel HD (2016). Model of the songbird nucleus HVC as a network of central pattern generators. Journal of Neurophysiology.

[bib7] Aronov D, Andalman AS, Fee MS (2008). A specialized forebrain circuit for vocal babbling in the juvenile songbird. Science.

[bib8] Beaulieu C, Kisvarday Z, Somogyi P, Cynader M, Cowey A (1992). Quantitative distribution of GABA-immunopositive and -immunonegative neurons and synapses in the monkey striate cortex (area 17). Cerebral Cortex.

[bib9] Beier T, Pape C, Rahaman N, Prange T, Berg S, Bock DD, Cardona A, Knott GW, Plaza SM, Scheffer LK, Koethe U, Kreshuk A, Hamprecht FA (2017). Multicut brings automated neurite segmentation closer to human performance. Nature Methods.

[bib10] Berning M, Boergens KM, Helmstaedter M (2015). SegEM: efficient image analysis for High-Resolution connectomics. Neuron.

[bib11] Binas J, Rutishauser U, Indiveri G, Pfeiffer M (2014). Learning and stabilization of winner-take-all dynamics through interacting excitatory and inhibitory plasticity. Frontiers in Computational Neuroscience.

[bib12] Bock DD, Lee WC, Kerlin AM, Andermann ML, Hood G, Wetzel AW, Yurgenson S, Soucy ER, Kim HS, Reid RC (2011). Network anatomy and in vivo physiology of visual cortical neurons. Nature.

[bib13] Braitenberg V, Schüz A (1998). Cortex: Statistics and Geometry of Neuronal Connectivity.

[bib14] Budd JM, Kovács K, Ferecskó AS, Buzás P, Eysel UT, Kisvárday ZF (2010). Neocortical axon arbors trade-off material and conduction delay conservation. PLoS Computational Biology.

[bib15] Cannon J, Kopell N, Gardner T, Markowitz J (2015). Neural sequence generation using spatiotemporal patterns of inhibition. PLoS Computational Biology.

[bib16] Cardin JA, Raksin JN, Schmidt MF (2005). Sensorimotor nucleus NIf is necessary for auditory processing but not vocal motor output in the avian song system. Journal of Neurophysiology.

[bib17] Coleman MJ, Vu ET (2005). Recovery of impaired songs following unilateral but not bilateral lesions of nucleus uvaeformis of adult zebra finches. Journal of Neurobiology.

[bib18] Colonnier M (1968). Synaptic patterns on different cell types in the different laminae of the cat visual cortex. an electron microscope study. Brain Research.

[bib19] Cragg B (1980). Preservation of extracellular space during fixation of the brain for electron microscopy. Tissue and Cell.

[bib20] de Wit J, Ghosh A (2016). Specification of synaptic connectivity by cell surface interactions. Nature Reviews Neuroscience.

[bib21] Denk W, Horstmann H (2004). Serial block-face scanning electron microscopy to reconstruct three-dimensional tissue nanostructure. PLoS Biology.

[bib22] Denk W, Webb WW (1990). Optical measurement of picometer displacements of transparent microscopic objects. Applied Optics.

[bib23] Dercksen VJ, Hege HC, Oberlaender M (2014). The filament editor: an interactive software environment for visualization, proof-editing and analysis of 3D neuron morphology. Neuroinformatics.

[bib24] Diesmann M, Gewaltig MO, Aertsen A (1999). Stable propagation of synchronous spiking in cortical neural networks. Nature.

[bib25] Dorkenwald S, Schubert PJ, Killinger MF, Urban G, Mikula S, Svara F, Kornfeld J (2017). Automated synaptic connectivity inference for volume electron microscopy. Nature Methods.

[bib26] Dutar P, Vu HM, Perkel DJ (1998). Multiple cell types distinguished by physiological, pharmacological, and anatomic properties in nucleus HVc of the adult zebra finch. Journal of Neurophysiology.

[bib27] Eccles J (1976). From electrical to chemical transmission in the central nervous system. Notes and Records of the Royal Society.

[bib28] Egger R, Dercksen VJ, Udvary D, Hege HC, Oberlaender M (2014). Generation of dense statistical connectomes from sparse morphological data. Frontiers in Neuroanatomy.

[bib29] Euler T, Hausselt SE, Margolis DJ, Breuninger T, Castell X, Detwiler PB, Denk W (2009). Eyecup scope--optical recordings of light stimulus-evoked fluorescence signals in the retina. Pflugers Archiv : European Journal of Physiology.

[bib30] Fee MS, Kozhevnikov AA, Hahnloser RH (2004). Neural mechanisms of vocal sequence generation in the songbird. Annals of the New York Academy of Sciences.

[bib31] Fiete IR, Senn W, Wang CZ, Hahnloser RH (2010). Spike-time-dependent plasticity and heterosynaptic competition organize networks to produce long scale-free sequences of neural activity. Neuron.

[bib32] Gibb L, Gentner TQ, Abarbanel HD (2009). Inhibition and recurrent excitation in a computational model of sparse bursting in song nucleus HVC. Journal of Neurophysiology.

[bib33] Glauert AM, Lewis PR (2014). Biological Specimen Preparation for Transmission Electron Microscopy.

[bib34] Goldin MA, Alonso LM, Alliende JA, Goller F, Mindlin GB (2013). Temperature induced syllable breaking unveils nonlinearly interacting timescales in birdsong motor pathway. PLoS One.

[bib35] Gray EG (1959). Electron microscopy of synaptic contacts on dendrite spines of the cerebral cortex. Nature.

[bib36] Hahnloser RH, Kozhevnikov AA, Fee MS (2002). An ultra-sparse code underlies the generation of neural sequences in a songbird. Nature.

[bib37] Hamaguchi K, Mooney R (2012). Recurrent interactions between the input and output of a songbird cortico-basal ganglia pathway are implicated in vocal sequence variability. Journal of Neuroscience.

[bib38] Hamaguchi K, Tanaka M, Mooney R (2016). A distributed recurrent network contributes to temporally precise vocalizations. Neuron.

[bib39] Harvey CD, Coen P, Tank DW (2012). Choice-specific sequences in parietal cortex during a virtual-navigation decision task. Nature.

[bib40] Helmstaedter M, Briggman KL, Denk W (2011). High-accuracy neurite reconstruction for high-throughput neuroanatomy. Nature Neuroscience.

[bib41] Helmstaedter M (2013). Cellular-resolution connectomics: challenges of dense neural circuit reconstruction. Nature Methods.

[bib42] Hopfield JJ, Tank DW (1985). Neural computation of decisions in optimization problems. Biological Cybernetics.

[bib43] Januszewski M, Maitin-Shepard J, Li P, Kornfeld J, Denk W, Jain V (2016). Flood-Filling networks. ArXiv eE-1Prints1611.

[bib44] Jiang X, Shen S, Cadwell CR, Berens P, Sinz F, Ecker AS, Patel S, Tolias AS (2015). Principles of connectivity among morphologically defined cell types in adult neocortex. Science.

[bib45] Jin DZ, Ramazanoğlu FM, Seung HS (2007). Intrinsic bursting enhances the robustness of a neural network model of sequence generation by avian brain area HVC. Journal of Computational Neuroscience.

[bib46] Karnovsky MJ (1971). Use of ferrocyanide-reduced osmium tetroxide in electron microscopy. Proc11th Annu Mtg Am Soc Cell Biol.

[bib47] Karsh B (2016). Github.

[bib48] Kasthuri N, Hayworth KJ, Berger DR, Schalek RL, Conchello JA, Knowles-Barley S, Lee D, Vázquez-Reina A, Kaynig V, Jones TR, Roberts M, Morgan JL, Tapia JC, Seung HS, Roncal WG, Vogelstein JT, Burns R, Sussman DL, Priebe CE, Pfister H, Lichtman JW (2015). Saturated reconstruction of a volume of neocortex. Cell.

[bib49] Komai S, Denk W, Osten P, Brecht M, Margrie TW (2006). Two-photon targeted patching (TPTP) in vivo. Nature Protocols.

[bib50] Kornfeld J (2017a). Github.

[bib51] Kornfeld J (2017b). Github.

[bib52] Kosche G, Vallentin D, Long MA (2015). Interplay of inhibition and excitation shapes a premotor neural sequence. Journal of Neuroscience.

[bib53] Kozhevnikov AA, Fee MS (2007). Singing-related activity of identified HVC neurons in the zebra finch. Journal of Neurophysiology.

[bib54] Kubota M, Taniguchi I (1998). Electrophysiological characteristics of classes of neuron in the HVc of the zebra finch. Journal of Neurophysiology.

[bib55] Li M, Greenside H (2006). Stable propagation of a burst through a one-dimensional homogeneous excitatory chain model of songbird nucleus HVC. Physical Review. E, Statistical, Nonlinear, and Soft Matter Physics.

[bib56] Long MA, Fee MS (2008). Using temperature to analyse temporal dynamics in the songbird motor pathway. Nature.

[bib57] Long MA, Jin DZ, Fee MS (2010). Support for a synaptic chain model of neuronal sequence generation. Nature.

[bib58] Lundqvist M, Rose J, Herman P, Brincat SL, Buschman TJ, Miller EK (2016). Gamma and beta bursts underlie working memory. Neuron.

[bib59] Lynch GF, Okubo TS, Hanuschkin A, Hahnloser RH, Fee MS (2016). Rhythmic Continuous-Time coding in the songbird analog of vocal motor cortex. Neuron.

[bib60] Markowitz JE, Liberti WA, Guitchounts G, Velho T, Lois C, Gardner TJ (2015). Mesoscopic patterns of neural activity support songbird cortical sequences. PLOS Biology.

[bib61] McGuire BA, Gilbert CD, Rivlin PK, Wiesel TN (1991). Targets of horizontal connections in macaque primary visual cortex. The Journal of Comparative Neurology.

[bib62] Mello GB, Soares S, Paton JJ (2015). A scalable population code for time in the striatum. Current Biology : CB.

[bib63] Mooney R, Prather JF (2005). The HVC microcircuit: the synaptic basis for interactions between song motor and vocal plasticity pathways. Journal of Neuroscience.

[bib64] Mooney R (2000). Different subthreshold mechanisms underlie song selectivity in identified HVc neurons of the zebra finch. Journal of Neuroscience.

[bib65] Mostafa H, Indiveri G (2014). Sequential activity in asymmetrically coupled winner-take-all circuits. Neural Computation.

[bib66] Narayanan RT, Egger R, Johnson AS, Mansvelder HD, Sakmann B, de Kock CP, Oberlaender M (2015). Beyond columnar organization: cell type- and target Layer-Specific principles of horizontal axon projection patterns in rat vibrissal cortex. Cerebral Cortex.

[bib67] Nixdorf-Bergweiler B, Bischof H-J (2007). A Stereotaxic Atlas of the Brain of the Zebra Finch, Taeniopygia Guttata.

[bib68] Nottebohm F, Kelley DB, Paton JA (1982). Connections of vocal control nuclei in the canary telencephalon. The Journal of Comparative Neurology.

[bib69] Nottebohm F, Stokes TM, Leonard CM (1976). Central control of song in the canary, serinus canarius. The Journal of Comparative Neurology.

[bib70] Oberlaender M, Broser PJ, Sakmann B, Hippler S (2009). Shack-Hartmann wave front measurements in cortical tissue for deconvolution of large three-dimensional mosaic transmitted light brightfield micrographs. Journal of Microscopy.

[bib71] Oberlaender M, Bruno RM, Sakmann B, Broser PJ (2007). Transmitted light brightfield mosaic microscopy for three-dimensional tracing of single neuron morphology. Journal of Biomedical Optics.

[bib72] Pastalkova E, Itskov V, Amarasingham A, Buzsáki G (2008). Internally generated cell assembly sequences in the rat hippocampus. Science.

[bib73] Peters A (2002). Examining neocortical circuits: some background and facts. Journal of Neurocytology.

[bib74] Peters AJ, Chen SX, Komiyama T (2014). Emergence of reproducible spatiotemporal activity during motor learning. Nature.

[bib75] Picardo MA, Merel J, Katlowitz KA, Vallentin D, Okobi DE, Benezra SE, Clary RC, Pnevmatikakis EA, Paninski L, Long MA (2016). Population-Level representation of a temporal sequence underlying song production in the zebra finch. Neuron.

[bib76] Pinault D (1996). A novel single-cell staining procedure performed in vivo under electrophysiological control: morpho-functional features of juxtacellularly labeled thalamic cells and other central neurons with biocytin or neurobiotin. Journal of Neuroscience Methods.

[bib77] Pologruto TA, Sabatini BL, Svoboda K (2003). ScanImage: flexible software for operating laser scanning microscopes. Biomedical Engineering Online.

[bib78] Rajan K, Harvey CD, Tank DW (2016). Recurrent network models of sequence generation and memory. Neuron.

[bib79] Rees CL, Moradi K, Ascoli GA (2017). Weighing the evidence in Peters' Rule: does neuronal morphology predict connectivity?. Trends in Neurosciences.

[bib80] Reiner A, Veenman CL, Medina L, Jiao Y, Del Mar N, Honig MG (2000). Pathway tracing using biotinylated dextran amines. Journal of Neuroscience Methods.

[bib81] Roberts TF, Klein ME, Kubke MF, Wild JM, Mooney R (2008). Telencephalic neurons monosynaptically link brainstem and forebrain premotor networks necessary for song. Journal of Neuroscience.

[bib82] Schalek R, Lee D, Kasthuri N, Peleg A, Jones T, Kaynig V, Haehn D, Pfister H, Cox D, Lichtman JW (2016). Imaging a 1 mm 3 volume of rat cortex using a MultiBeam SEM. Microscopy and Microanalysis.

[bib83] Scharff C, Kirn JR, Grossman M, Macklis JD, Nottebohm F (2000). Targeted neuronal death affects neuronal replacement and vocal behavior in adult songbirds. Neuron.

[bib84] Scheffer LK, Karsh B, Vitaladevun S (2013). Automated alignment of imperfect EM images for neural reconstruction. arXiv.

[bib85] Scott BB, Gardner T, Ji N, Fee MS, Lois C (2012). Wandering neuronal migration in the postnatal vertebrate forebrain. Journal of Neuroscience.

[bib86] Seligman AM, Wasserkrug HL, Hanker JS (1966). A new staining method (OTO) for enhancing contrast of lipid--containing membranes and droplets in osmium tetroxide--fixed tissue with osmiophilic thiocarbohydrazide(TCH). The Journal of Cell Biology.

[bib87] Seung HS (2009). Reading the book of memory: sparse sampling versus dense mapping of connectomes. Neuron.

[bib88] Sholl DA (1953). Dendritic organization in the neurons of the visual and motor cortices of the cat. Journal of Anatomy.

[bib89] Stepanyants A, Martinez LM, Ferecskó AS, Kisvárday ZF (2009). The fractions of short- and long-range connections in the visual cortex. PNAS.

[bib90] Usher M, McClelland JL (2001). The time course of perceptual choice: the leaky, competing accumulator model. Psychological Review.

[bib91] Vallentin D, Long MA (2015). Motor origin of precise synaptic inputs onto forebrain neurons driving a skilled behavior. Journal of Neuroscience.

[bib92] Vu ET, Mazurek ME, Kuo YC (1994). Identification of a forebrain motor programming network for the learned song of zebra finches. Journal of Neuroscience.

[bib93] Walton J (1979). Lead aspartate, an en bloc contrast stain particularly useful for ultrastructural enzymology. The Journal of Histochemistry and Cytochemistry : Official Journal of the Histochemistry Society.

[bib94] Wang N, Hurley P, Pytte C, Kirn JR (2002). Vocal control neuron incorporation decreases with age in the adult zebra finch. Journal of Neuroscience.

[bib95] Wild JM, Williams MN, Howie GJ, Mooney R (2005). Calcium-binding proteins define interneurons in HVC of the zebra finch (Taeniopygia guttata). The Journal of Comparative Neurology.

[bib96] Yildiz IB, Kiebel SJ (2011). A hierarchical neuronal model for generation and online recognition of birdsongs. PLoS Computational Biology.

